# Early-life histories, morphological development, and dichotomous keys of seven wild mouth-brooding fighting fish species (Actinopterygii, Osphronemidae)

**DOI:** 10.3897/zookeys.1281.184196

**Published:** 2026-06-09

**Authors:** Santi Poungcharean, Idsariya Wudtisin, Soranuth Sirisuay, Phongchate Pichitkul, Sommai Janekitkarn

**Affiliations:** 1 Department of Fishery Biology, Faculty of Fisheries, Kasetsart University, Chatuchak, Bangkok, Thailand Department of Fishery Biology, Faculty of Fisheries, Kasetsart University Bangkok Thailand; 2 Department of Aquaculture, Faculty of Fisheries, Kasetsart University, Chatuchak, Bangkok, Thailand Department of Aquaculture, Faculty of Fisheries, Kasetsart University Bangkok Thailand

**Keywords:** *
Betta
*, fish larvae, Identification, morphological development, mouth-brooding fighting fish, ontogeny

## Abstract

Within the family Osphronemidae, mouth-brooding fighting fishes are small, air-breathing fish species that hold their eggs and offspring in their buccal cavities as a parental care behavior, usually found in running waters and distributed in the wild throughout Southeast Asia. This study aims to describe the morphological development and generate identification keys for the larval and juvenile stages of seven wild mouth-brooding fighting fish species found in Thailand, i.e., *Betta apollon*, *B. ferox*, *B. pallida*, *B. pi*, *B. prima*, *B. pugnax*, and *B. simplex* from the wild. The broodstocks were collected from type localities or based on characteristics that most closely matched each species description, with healthy fishes selected and breeding continued for our size-series collection, including *B. simplex*, a previous study. The results showed that mouth-brooders released their offspring when developed to the post-flexion stage within 11–12 (mode = 11) days after fertilization (DAF), except *B. pi*, which took 18–20 DAF, and the post-flexion larva developed to the juvenile stage within 18–30 days after release. The main characteristics of the new-release post-flexion larva were an oblong and depressed body, a large head, an oval to rounded eye, and rays where the caudal fin began to develop, as well as having fully developed ventral fins with two or three dorsal, central, and ventral stripes and a caudal spot. Myomere numbers and fin rays differed among species across a range of 8–10 dorsal, 10–13 pectoral, 6 ventral, 24–31 anal, and 10–13 caudal fin rays. Diagnostic characters were selected to create a dichotomous identification key, with an illustration provided. In terms of taxonomy, the different larval stages also differed in pigmentation patterns among species, with pigmentation patterns on head (pre-orbital, sub-orbital, post-orbital, and sub-opercular bands) and longitudinal stripes on the side of the body able to be used to distinguish among different development stages as well as different species.

## Introduction

Bettas or fighting fishes, are small air-breathing fish in the family Osphronemidae, widely distributed in freshwater in Southeast Asia. A well-known species representing this genus is *Betta splendens*, or Siamese fighting fish, which aquaculturists have bred for fighting and ornamental purposes. Fighting fishes can be divided into two groups separated by parental care behavior, those who mouth-brood their eggs and offspring (mouth-brooding fighting fishes) and build bubble-nests for their eggs (bubble-nesting fighting fishes) (Schindler and Schmidt 2006; Panijpan et al. 2017, 2020). In Thailand, most mouth-brooding fighting fishes live in cold, running water, but habitat still varies between each species, e.g., *Betta apollon* Schindler & Schmidt, 2006, *B. ferox* (Schindler & Schmidt, 2006), and *B. pugnax* usually live in hill streams, *B. prima* Kottelat, 1994 and *B. pallida* Schindler & Schmidt, 2004 in small vegetative canals, *B. simplex* Kottelat, 1994 only in small lakes and creeks connected to limestone rivers, and *B. pi* Tan, 1998 only in southern Thai peat swamps (Panijpan et al. 2014). All these species, especially *B. simplex* and *B. pi*, have seen a trend of population decline in natural habitats caused by habitat degradation and commercial catching (Panijpan et al. 2014; Poungcharean and Limpivadhana 2022). The International Union for Conservation of Nature and Natural Resources (IUCN) has recently classified threatened status of *B. simplex* as critically endangered (CR), *B. pi* as endangered (EN), *B. prima* and *B. pugnax* as least concern (LC), and *B. ferox* and *B. pallida* as data deficient (DD) (IUCN 2025). Additionally, the Government Gazette of Thailand notifies that the Thai government has banned the export of *B. simplex* from the Kingdom of Thailand. (Ministry of Agriculture and Cooperatives, Government Gazette 2021).

Morphological description of larval fish is a database for reference in larval fish identification. Accurate species identification combined with quantitative studies of its populations in natural waters is beneficial for spawning ground and season. Quantitative studies in natural waters, combined with larval analysis, can help assess spawning grounds and seasons and provide comprehensive information on juvenile life, supporting aquaculture and conservation efforts. Additionally, it provides information on early-life histories, which enhances aquaculture and conservation efforts. Mouth-fighting fishes urgently require a reference description database, as they are a sensitive and threatened species. Attempts have been made to study the morphological development of mouth-brooding fighting fishes, but few reports are currently available. There is only one report on *B. simplex* by Poungcharean et al. (2025). While the bubble-nesting fighting group has been studied more extensively, as by Termvidchakorn (2005), who described the development of fish in the family Osphronemidae including two species of bubble-nesting fighting fish. At that time, the domesticated variants of *B. smaragdina* and *B. splendens* were described as representatives of the genus *Betta* and compared to other genera in the same family. Subsequently, Poungcharean and Limpivadhana (2022) succeeded in breeding and described the morphological development of all five wild bubble-nesting fighting fish species recorded in Thailand. Therefore, the morphological description of mouth-brooding fighting fishes should also be established for their breeding and conservation.

This study aimed to establish information on the morphological development, stage transformation, and early-life biology of seven mouth-brooding fighting fish species native to Thailand. We revealed the previous studies, especially *B. simplex*, and observed the representatives of Thai mouth-brooding fighting fishes for broodstocks that most closely matched the type specimen descriptions and then bred and reared them in captivity for our size-series collection. Larval specimen examinations measured the head, body, and fins and illustrated character and pigmentation. Additionally, we also generated a dichotomous key for identifying all seven species in their new-release and juvenile stages. The information provided in this study will deepen knowledge of the early-life histories of these mouth-brooding fighting fishes and beneficial for breeding conservation and management of their wild populations.

## Materials and methods

### Species collection and background

We have studied the morphological development of the seven species of mouth-brooding fighting fishes distributed in Thailand. Of all these, six species have been successfully bred in breeding captivities and deposit as a reference size-series specimen collection. The previously studied *B. simplex* by Poungcharean et al. (2025) was combined with additional examinations of reference specimens deposited at the Kasetsart University Museum of Fisheries, Faculty of Fisheries, Kasetsart University (**KUMF**). The dataset of all species was revised and matched for morphological descriptions and statistical analyses.

### Broodstock collection and breeding

**Figure 1.**
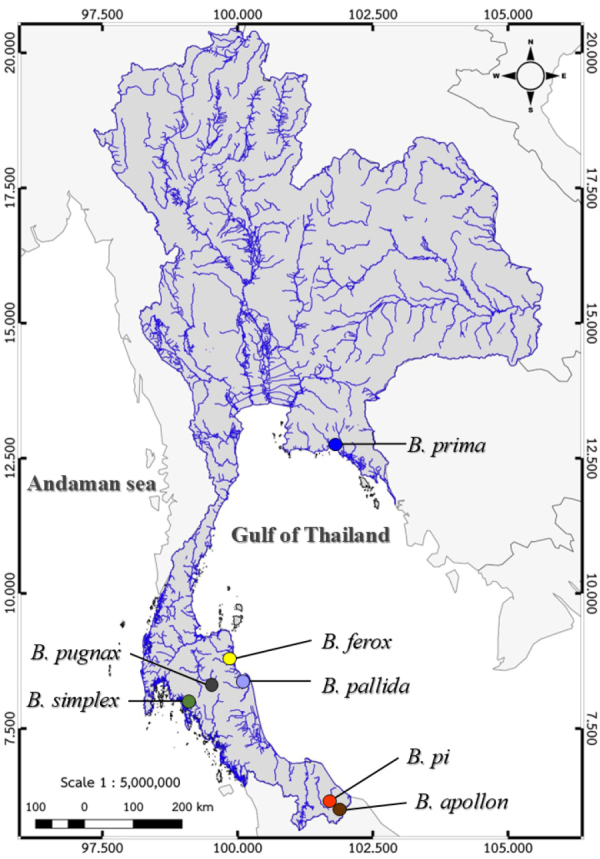
Location of the broodstock localities of seven wild mouth-brooding fighting fishes in Thailand.

Broodstocks of seven mouth-brooding bettas were collected either as close as possible to their type specimen locality or from fish most similar to the original descriptions. Mostly the fish species are distributed in southern Thailand, except *B. prima* which occurs in eastern Thailand. They were collected by dip net and “bung kee”, a local plastic basket with a handle. The range in approximate standard length in mm (SL), locality, and collection data (Fig. 1) of the species were as follows: 1. *Betta apollon* (37–62 mm SL)—Phru Toh Dang peat swamp, Bujok subdistrict, Su-gnai Kolok district, Narathiwat province (6°09'48"N, 101°54'41"E) (type specimen locality), 15 February 2021; 2. *B. ferox* (50–52 mm SL)—a hill stream of Tapan subdistrict, Sri Banphot district, Phatthalung province (7°37'36"N, 99°54'52"E), 5 May 2021; 3. *B. pallida* (31–36 mm SL)—an irrigation canal of Ronphibun subdistrict, Ronphibun district, Songkhla province (8°13'06"N, 99°49'48"E), 20 March 2021; 4. *B. pi* (60–66 mm SL)—Phru Toh Dang peat swamp, Su-gnai Padi subdistrict, Su-gnai Kolok district, Narathiwat province (6°12'22"N, 101°58'53"E) (holotype specimen locality), 16 February 2021; 5.); 5. *B. prima* (30–35 mm SL)—Klong Phlio canal, Phlio subdistrict, Muang district, Chanthaburi province (12°43'40"N, 102°11'09"E) (holotype specimen locality), 20 October 2020; 6. *B. pugnax* (38–40 mm SL)—Wang Pring hill stream, Thamyai subdistrict, Thungsong district, Nakhon Si Thammarat province (8°12'20"N, 99°45'01"E), 20 March 2021; 7. *B. simplex* refers to Poungcharean et al. (2025) as 30–34 mm SL—Klong Sra-Keaw creek, a connecting canal between a limestone river and Sra-Keaw Pond, Kao Thong subdistrict, Muang district, Krabi province (8°10'08"N, 98°48'28"E) (holotype specimen locality). Healthy and mature fishes were selected as broodstock and transported to the Laboratory of Ichthyology and Aquatic Ecology, Faculty of Fisheries, Kasetsart University.

Each species’ four or five broodstock couples were acclimatized and reared in 30 × 90 × 30 cm glass tanks. Submerged plants (*Anubius* spp.) and 2-cm diameter and 10-cm long PVC pipes were provided for shelter, and the fish were fed twice daily with adult artemia (*Artemia salina*) and grindal worm (*Enchytraeus buchholzi*). The broodstock tanks were placed in open-air conditions at a water temperature of 28–32 C°, except for *B. pi*, which naturally lives in cold water and was reared in a 25–27 C° temperature-controlled chiller fish tank. The broodstocks would mate in the early nighttime, and the parental males were observed every morning. The parental care of males can be observed through a bulging out of the buccal membrane (the anteroventral side of the lower lip), stopping eating, and the fish hiding in vegetation or PVC pipes. The first fertilized time was assumed to be the evening of the day before observing the males exhibiting parental care. Fertilized eggs develop and are expected to hatch within 7–8 days. During this period, we avoided interfering with the parental males to prevent the release or eating of their brooding eggs before the appropriate time. Then, the eighth-day parental males were isolated to avoid the offspring’s predation by the other fish. After release, the new offspring were fed water fleas (*Moina macrocopa*) and artemia nauplii (*Artemia salina*).

Various environmental parameters were recorded at the sampling sites for 3–5 replications per water body. Dissolved oxygen (DO) content, water temperature, pH, conductivity, and salinity were measured using a DO meter (YSI Pro 2030–Fondriest Environmental, Inc., Fairborn, Ohio, USA); turbidity was measured using a portable turbidity meter (Lamotte Model, 2020–Lamotte Company, Chestertown, USA). The water quality parameters required a laboratory, a water sample was collected and analyzed as follows: hardness, alkalinity, ammonia-nitrogen, nitrite-nitrogen, and orthophosphate-phosphorus were analyzed according to APHA et al. (2017) and Grasshoff (1976). Nitrate-nitrogen was analyzed using the cadmium reduction method.

### Specimen size series collection

Larval specimens were sampled for size-series collection according to Termvidchakorn (2005). However, complete size-series collection could not be carried out under low broodstock fecundity, and some reared larval fish always died during the nursing period. In these situations, 3–5 larval specimens of a series of different ages were required. Furthermore, specimen age was reported in both Days After Fertilization (DAF), counting the days from the first observed parental male, and Days After Release (DAR) counting from the first offspring releasing. At this stage, an 11-part size-series comprising offspring at 0, 3, 6, 9, 12, 15, 20, 25, 30, 35, and 40 DAR was constructed. The specimens were anesthetized using an overdose of eugenol solution before being preserved in 4% neutralized formaldehyde and deposited in the Kasetsart University Museum of Fisheries (KUMF7780–7784, and 7788). For the KUMF collection of *B. simplex* specimens (KUMF 7771), a species previously described by Poungcharean et al. (2025), *B. simplex*, was revealed and additionally examined to create a dataset of morphological characters and statistical testing with other species.

### Specimen examination and data analysis

**Figure 2.**
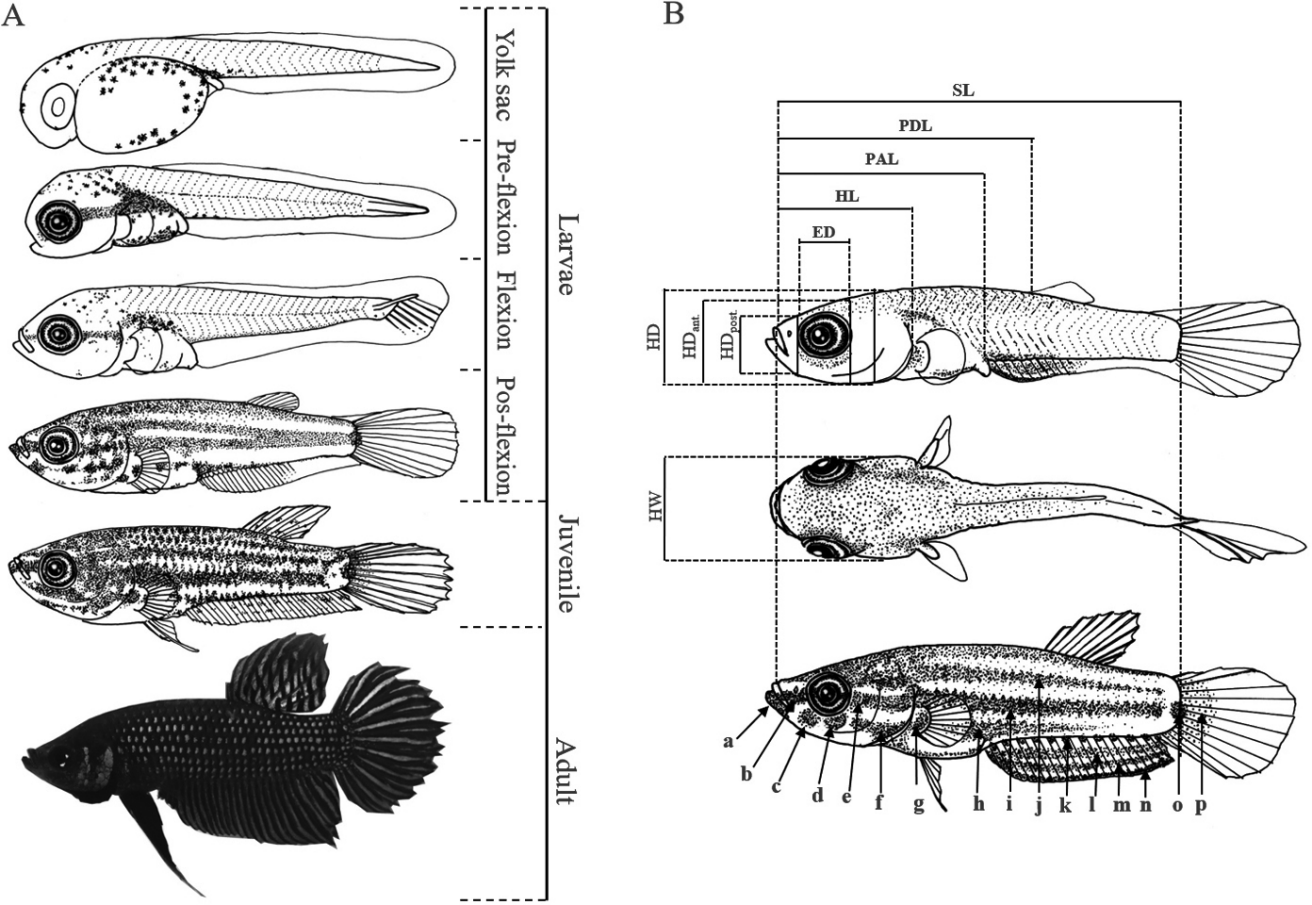
Developmental stages of fighting fish. **A**. General illustration of mouth-brooding fighting fish development stages; **B**. Illustration showing morphometric characteristics and dark pigmentation. Abbreviations of body dimensions: SL = standard length, BD = body depth, HL = head length, HD = head depth, HD_ant_ = head depth at anterior edge of eye, HD_post_ = head depth at posterior edge of eye, HW = head width, ED = eye diameter, PAL = pre-anal length, PDL = pre-dorsal length. Abbreviations for pigmentation patterns: a = chin band, b = pre-orbital band, c = first sub-orbital band, d = second sub-orbital band, e = first post-orbital band, f = second post-orbital band, g = pectoral fin base band, h = ventral stripe, i = central stripe, j = dorsal stripe, k = anal fin base band, l = inner sub-marginal stripe, m = outer sub-marginal stripe, n = marginal stripe, o = caudal spot, p = scattering caudal fin spots.

The developmental stages were classified according to Balon (1985) (Fig. 2A). Morphological measurements followed Leis and Carson-Edwart (2000), with photographic specimens measured with a scale bar under a stereomicroscope. Nine body measurements were examined in this study, i.e., standard length (**SL**), head length (**HL**), head depth (**HD**), head depth at eye anterior (**HD_ant_**), head depth at eye posterior (**HD_post_**), head width (**HW**), eye diameter (**ED**), pre-dorsal length (**PDL**), pre-anal length (**PAL**), and body depth (**BD**) (Fig. 2B).

The body dimensions were expressed as percentages of standard length (%SL) when referring to the body and head, percentages of head length (%HL) when referring to the eye and snout, and percentages of head depth (%HD) for head profile shape. Myomeres were counted in the post-flexion stage when the bodies of specimens were transparent, and fin rays were counted in juvenile specimens when fins were completely developed. Melanophore patterns (pigmentation patterns) were recorded, with the pattern types comprising ‘bands’ for dark pigments positioned on head regions, ‘stripes’ for longitudinal dark lines along the sides of the body and fin, and ‘caudal spot’ for a dark band at the base of caudal fin (Fig. 2B). The data on rearing water qualities and specimen body dimensions are presented as mean and standard deviation, including meristic features such as myomere and fin ray count, which are presented with mode, minimum, and maximum values.

Morphological characters of head and body dimensions of the seven mouth-brooding fighting fishes were tested for variability for species classification. Six morphometric characters (i.e., head length, body depth, preanal length, head width, eye diameter, and head depth) of the new-release post-flexion stage and eight meristic characters (i.e., total myomere, preanal myomere, postanal myomere, dorsal fin ray, pectoral fin ray, pelvic fin ray, anal fin ray, and caudal fin ray) of the juvenile stage were performed by using Principal Component Analysis (PCA). The PCA was performed by using the paleontological statistics software package for education and data analysis (program PAST) (Hammer et al. 2001).

## Results

### Reproduction and rearing environmental conditions

**Figure 3.**
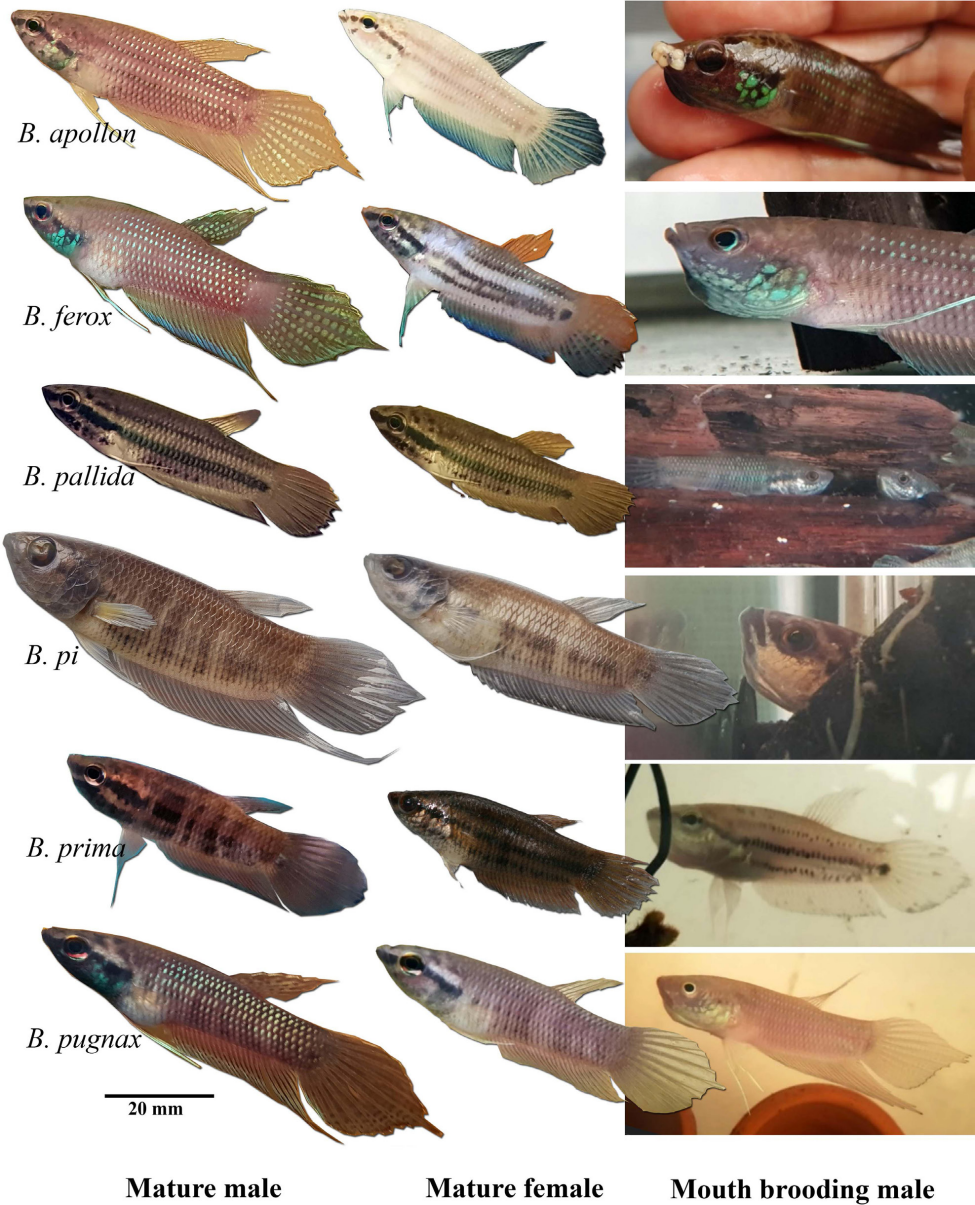
Broodstocks of six wild mouth-brooding fighting fishes for specimen size-series collection.

All broodstocks taken from the wild were successful at breeding in laboratory conditions with a mean and standard deviation (SD) of water temperature of 30.70 (SD = 2.26) °C, dissolved oxygen of 5.74 (SD = 0.53) mg·L^−1^, and pH of 7.12 (SD = 0.11). The broodstocks mostly acclimatized and sexually developed to their mature stage within 2–4 months, and the fish were ready to spawn, except for *Betta pi*, which takes longer (4–5 months) because it inhabits a specific type of low-pH and -temperature water, with *B. pi*’s breeding conditions having a water temperature of 26.15 (SD = 0.59) °C, dissolved oxygen of 5.50 (SD = 0.71) mg·L^−1^, and pH of 6.1 (SD = 0.9). A comparison of other water qualities between the *B. pi* environment, which is a peat and limestone swamp condition, and those of the other mouth-brooding fighting fishes is shown in Table 1. The mature males could be distinguished from females by their shinier body color and longer anal fin end tip, with mature females characterized by a pale-yellow bulging belly and a genital papilla in front of the anal fin base (Fig. 3).

**Table 1.** Comparison of water qualities (mean ± standard deviation) between other mouth-brooding fighting fishes and *Betta pi* broodstock in breeding captivity.

Water qualities	Breeding condition	
	Other mouth-brooders	*B. pi*
Water temperature (°C)	30.70 ± 2.26	26.15 ± 0.59
Dissolved oxygen (mg·L^−1^)	5.74 ± 0.53	5.50 ± 0.71
Turbidity (NTU)	118.67 ± 6.11	125.10 ± 5.90
pH	7.12 ± 0.11	6.10 ± 0.9
Salinity (ppt)	0.1	0.1
Conductivity (µS·cm^−1^)	301 ± 10	188 ± 12
Hardness (mg·L^−1^ as CaCO_3_)	69 ± 24	147 ± 5
Alkalinity (mg·L^−1^ as CaCO_3_)	28 ± 3	31 ± 2
Ammonia–nitrogen (mg·L^−1^)	0.2467 ± 0.0035	0.2313 ± 0.0029
Nitrite–nitrogen (mg·L^−1^)	0.0104 ± 0.0009	0.0137 ± 0.0032
Orthophosphate–phosphorus (mg·L^−1^)	0.0177 ± 0.0052	0.0178 ± 0.0008

Broodstock mating was observed during the early-to-mid-rainy season (June–August 2021) for *B. ferox*, *B. pugnax*, and *B. apollon*; mid-to-late rainy season (September–October 2021) for *B. simplex*; cold season (November–December 2021) for *B. pi*; and throughout the year, except November to December, for *B. pallida* and *B. prima*. All parental males successfully mated and continued to perform parental care for their offspring until the completion of size-series collection. The couples were easily observed due to their separation from schools to hide under their provided shelters (submerged plants and PVC pipes). This paired mating behavior was similar to that of bubble-nesting fighting fishes but did not involve bubble nest building, with males instead gathering and carrying fertilized eggs in their mouth cavity.

### Growth and stage transformation

Fertilized eggs were safely developed in the mouth cavity of parental males, with the incubation period and offspring release mostly occurring within 11–12 DAF (mostly 11); however, *B. pi* showed a longer incubation period of 18–20 DAF. Fecundity, which refers to the number of first-released offspring, was 35–90 offspring per crop. To track morphological development, a 10–12-specimen series was registered as a museum referent collection at the KUMF. In the first post-release stage, the various species of offspring develop to their post-flexion stage with a variety of average sizes between 3.73 and 5.60 mm SL. The smallest species was *B. prima* (3.73 [SD = 0.00 mm SL], *n* = 3), and the largest was *B. pi* (5.60 [SD = 0.12] mm SL, *n* = 5). At this stage, the yolk-sac is already absorbed; caudal fin rays are developed; pectoral, dorsal, and anal fin rays appear only as fin-folds or in early development; and pelvic buds and scales are not yet developed. The fish develop to the juvenile stage within 18–30 DAF, with *B. pi* being the fastest-developing. The age, developmental stage, and body size of each species are shown in Table 2.

**Table 2.** Fecundity, age at each developmental stage, and body size of six reared mouth-brooding fighting fishes.

Species	Expected age first release (DAF)	Fecundity (offsprings per crop)	Size at first release (mm SL)	Age at entry into juvenile (DAR)	Size at entry into juvenile (mm SL)
*B. apollon*	11 (mode = 11, *n* = 2)	35–45 (*n* = 2)	4.76 ± 0.01 (*n* = 3)	24	11.30 ± 0.53 (*n* = 3)
*B. ferox*	11	40 (*n* = 1)	4.63 ± 0.01 (*n* = 4)	24	11.30 (*n* = 1)
*B. pallida*	11-12 (mode = 11, *n* = 4)	55–80 (*n* = 5)	4.98 ± 0.00 (*n* = 5)	21	10.32 ± 0.38 (*n* = 3)
*B. pi*	18-20	70–80 (*n* = 2)	5.60 ± 0.12 (*n* = 12)	18	14.55 ± 0.82 (*n* = 5)
*B. prima*	11 (mode = 11, *n* = 2)	75–90 (*n* = 2)	3.73 ± 0.00 (*n* = 3)	25	10.38 ± 0.30 (*n* = 3)
*B. pugnax*	11	75 (*n* = 1)	4.65 ± 0.00 (*n* = 3)	25	11.53 ± 0.75 (*n* = 3)

### Statistical analysis

**Figure 4.**
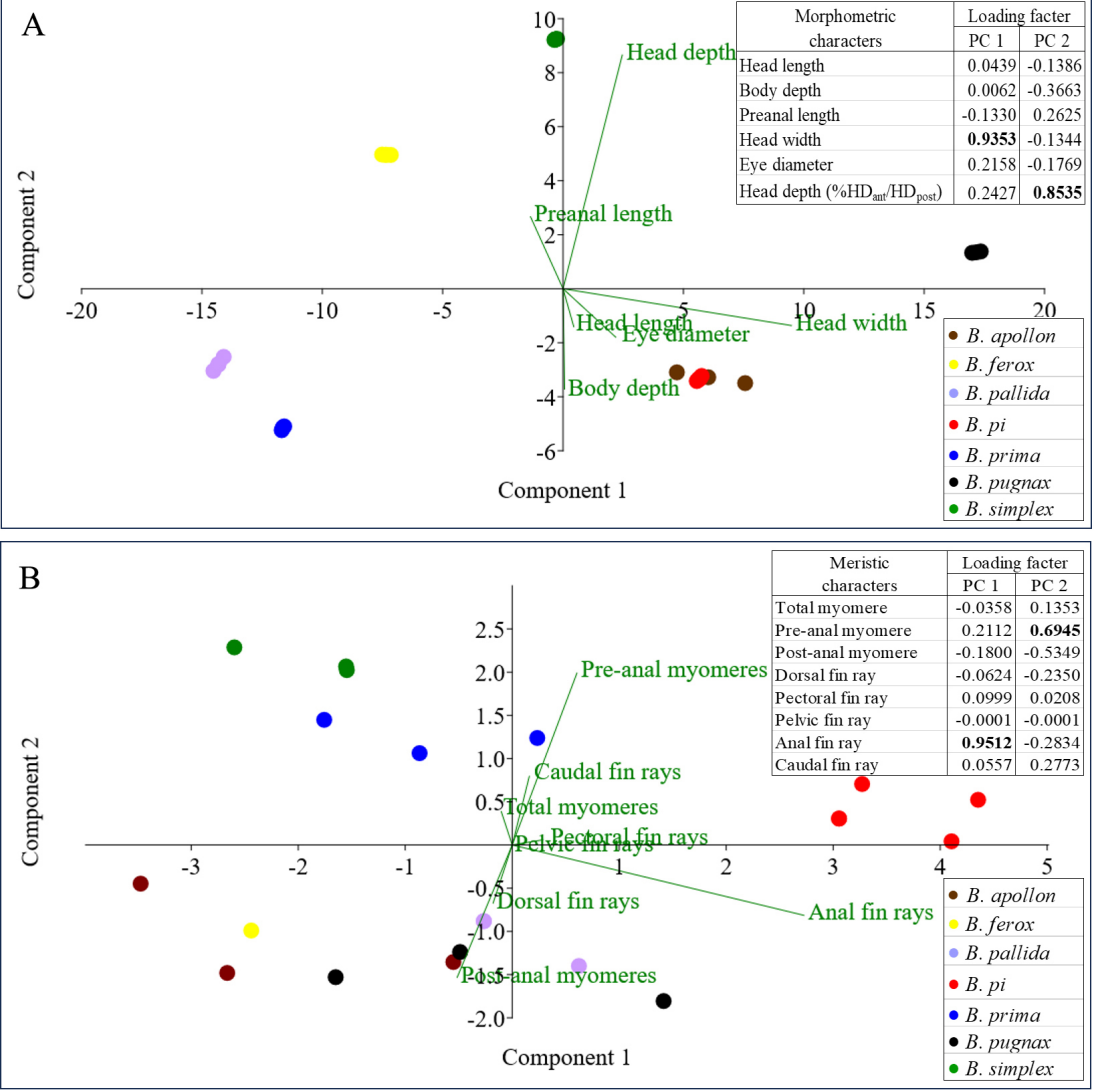
Scatter plot with loading scores of PC1 and PC2 of morphological characters of different development stages of seven mouth-brooding fighting fishes. Morphometric characters of the new release pre-flexion stage (**A**) and myomere count of the new release pre-flexion stage and fin ray count of the juvenile stage (**B**). Bolded numbers in the PC1 and PC2 columns indicate high loading factors.

According to the summarized of morphometric and meristic characters in Table 3, the flexibility of morphological characters of seven species of juvenile fish was performed by PCA, based on the available data at each developmental stage. At the new release pre-flexion stage, the fin ray has not developed; head and body dimensions were considered for analysis as morphometric characters. According to the scatter plot with loading scores in Fig. 4, the loading score PC1 was high for head width (0.9353), while PC2 was high for head depth (0.8535) (Fig. 4A). Myomere counts of the pre-flexion stage and completely developed fin ray counts of the juvenile stage were used as meristic characters. The meristic characters have a high loading score on PC1 by anal fin ray (0.9512), while PC2 is high by pre-anal myomere (0.6945) (Fig. 4B).

**Table 3.** Morphometric characters and number of myomeres and fin rays for seven wild mouth-brooding fighting fishes. “*n*” in parentheses is the number of specimen series of new release post-flexion stage + the number of specimen series entering the juvenile stage.

Characters	*B. apollon* (*n* = 3 + 3)		*B. ferox* (*n* = 4 + 1)		*B. pallida* (*n* = 5 + 3)		*B. pi* (*n* = 12 + 5)		*B. prima* (*n* = 3 + 3)		*B. pugnax* (*n* = 3 + 3)		*B. simplex* (*n* = 6 + 3)*	
	Min.-Max.	Mean ± SD	Min.-Max.	Mean	Min.-Max.	Mean ± SD	Min.-Max.	Mean ± SD	Min.-Max	Mean ± SD	Min.-Max.	Mean ± SD	Min.-Max.	Mean ± SD
New-release post-flexion stage														
Standard length (mm)	4.75–4.76	4.76 ± 0.01	4.63–4.64	4.63 ± 0.01	4.98–4.98	4.98 ± 0.00	5.44–5.72	5.60 ± 0.12	3.73–3.73	3.73 ± 0.00	4.65–4.65	4.65 ± 0.00	4.39–4.40	4.39 ± 0.10
Head length (%SL)	33.48–33.51	33.50 ± 0.01	32.43–32.44	32.44 ± 0.01	33.33–33.33	33.33 ± 0.00	33.39–33.62	33.48 ± 0.10	32.33–32.36	32.35 ± 0.01	34.33–34.45	34.39 ± 0.06	31.18–31.21	31.19 ± 0.02
Body depth (%SL)	25.31–25.52	25.44 ± 0.08	20.25–20.28	20.27 ± 0.02	23.41–23.67	23.53 ± 0.11	23.48–23.56	23.53 ± 0.04	25.42–25.46	25.44 ± 0.02	22.52–22.55	22.54 ± 0.02	19.67–19.70	19.68 ± 0.02
Pre-anal length (%SL)	49.89–50.10	50.00 ± 0.01	53.10–53.23	53.15 ± 0.10	51.88–52.06	51.98 ± 0.08	49.03–49.15	49.11 ± 0.05	54.34–54.44	54.39 ± 0.04	50.98–51.07	51.03 ± 0.05	54.07–54.18	54.13 ± 0.06
Head width (%HD)	122.46–124.38	123.81 ± 0.08	108.58–108.91	108.70 ± 0.17	103.97–104.20	104.08 ± 0.09	121.64–121.77	121.74 ± 0.07	107.39–107.45	107.41 ± 0.03	133.92–134.26	134.09 ± 0.17	115.18–115.28	115.23 ± 0.05
Eye diameter (%HL)	40.46–40.93	40.63 ± 0.02	41.55–41.79	41.67 ± 0.12	38.09–38.33	38.24 ± 0.10	47.03–47.10	47.06 ± 0.03	39.98–40.05	40.00 ± 0.03	41.64–41.70	41.67 ± 0.03	41.14–41.21	41.18 ± 0.04
Head Depth_ant_ (%HD_post_)	81.37–81.49	81.43 ± 0.05	85.53–85.59	85.56 ± 0.03	76.58–77.33	76.92 ± 0.31	81.69–81.98	81.82 ± 0.12	74.88–75.06	75.00 ± 0.08	87.21–87.34	87.27 ± 0.07	90.71–90.77	90.74 ± 0.03
Entry into juvenile stage														
Standard length (mm)	10.78–11.83	11.30 ± 0.53	11.30–11.30	11.3	9.97–10.68	10.32 ± 0.38	14.00–15.71	14.55 ± 0.82	10.04–10.75	10.38 ± 0.30	10.69–12.51	11.53 ± 0.75	11.06–12.29	11.68 ± 0.62
Head length (%SL)	34.45–34.64	34.55 ± 0.10	33.33–33.33	33.33	34.24–34.37	34.30 ± 0.05	33.41–33.48	33.45 ± 0.04	35.19–35.25	35.22 ± 0.02	36.48–36.54	36.51 ± 0.03	34.60–34.65	34.63 ± 0.03
Pre-dorsal length (%SL)	63.93–64.07	64.00 ± 0.07	69.05–69.05	69.05	72.12–72.30	72.20 ± 0.08	65.39–65.49	65.44 ± 0.05	71.39–71.44	71.43 ± 0.02	65.41–65.53	65.48 ± 0.05	69.58–69.63	69.61 ± 0.03
Pre-anal length (%SL)	54.49–54.60	54.55 ± 0.06	55.56–55.56	55.56	51.87–52.06	51.99 ± 0.09	55.12–55.17	55.15 ± 0.03	51.78–51.95	51.88 ± 0.07	55.9–56.02	55.95 ± 0.05	54.69–54.84	54.77 ± 0.08
Head width (%HD)	71.40–71.40	71.43 ± 0.03	67.24–67.24	67.24	64.67–64.75	64.71 ± 0.03	70.70–.70.77	70.73 ± 0.04	62.69–62.81	62.75 ± 0.05	74.27–74.35	74.31 ± 0.03	68.65–68.70	68.67 ± 0.03
Meristics of juvenile stage	Min.-Max.	Mode	Min.-Max	Mode	Min.-Max	Mode	Min.-Max	Mode	Min.-Max.	Mode	Min.-Max.	Mode	Min.-Max	Mode
Total myomeres	31–31	31	31–31	31	30–30	30	30–31	31	31–32	32	31–31	31	31–31	31
Pre-anal myomeres	9–10	9	9–10	9	9–10	9	11–12	11	11–12	11	9–9	9	11–11	11
Post-anal myomeres	21–22	22	22–22	22	21–21	21	19–20	20	20–21	21	22–22	22	20–20	20
Dorsal fin rays	9–10	10	8–9	8	8–9	8	8–9	8	8–9	8	11–12	9	8–9	8
Pectoral fin rays	10–11	11	11–12	12	11–12	12	11–13	11	11–12	12	11–12	12	11–12	12
Pelvic fin rays	6–6	6	6–6	6	6–6	6	6–6	6	6–6	6	6–6	6	6–6	6
Anal fin rays	24–27	25	24–25	25	26–28	27	30–31	30	25–27	26	26–29	27	24–25	25
Caudal fin rays	10–10	10	10	10	11–12	11	11–11	11	10–11	11	10–12	12	12–13	12

Remarks: * the revised data is from Poungcharean et al. (2025) and includes an examination of the reference specimen collection.

### Morphological description of seven mouth-brooding fighting fishes

We describe the morphology and produced illustrations of the offspring, comprising new-release post-flexion and juvenile stages. Based on the KUMF reference specimen series, the new-release post-flexion larvae had oblong and compressed posterior body parts, large and depressed heads with a proportion of head length greater than 100%HD, their guts coiled and opened in the middle of their bodies, and total myomere counts numbered 31 or 32 (mostly 31). On the other hand, the juvenile stage fish had similar fin ray counts, ranging from 8–10 dorsal, 11–13 pectoral, 6 pelvic, 24–31 anal, and 10–13 caudal fin rays. As for other distinguishing features of each species, melanophore pigmentation, head and eye shape, myomere numbers, and anal fin rays were advantageous for generating an identification key (dichotomous key). The morphological descriptions and illustrations of the development stages were selected from specimen age series to represent each developmental stage older than the data in Table 2, as we aim to select the size series with complete and clearly visible fin ray development and melanophore pigmentation. Morphological descriptions of the seven mouth-brooding fighting fishes are as follows:

### *Betta apollon* Schindler & Schmidt, 2006 (KUMF 7782)

**Figure 5.**
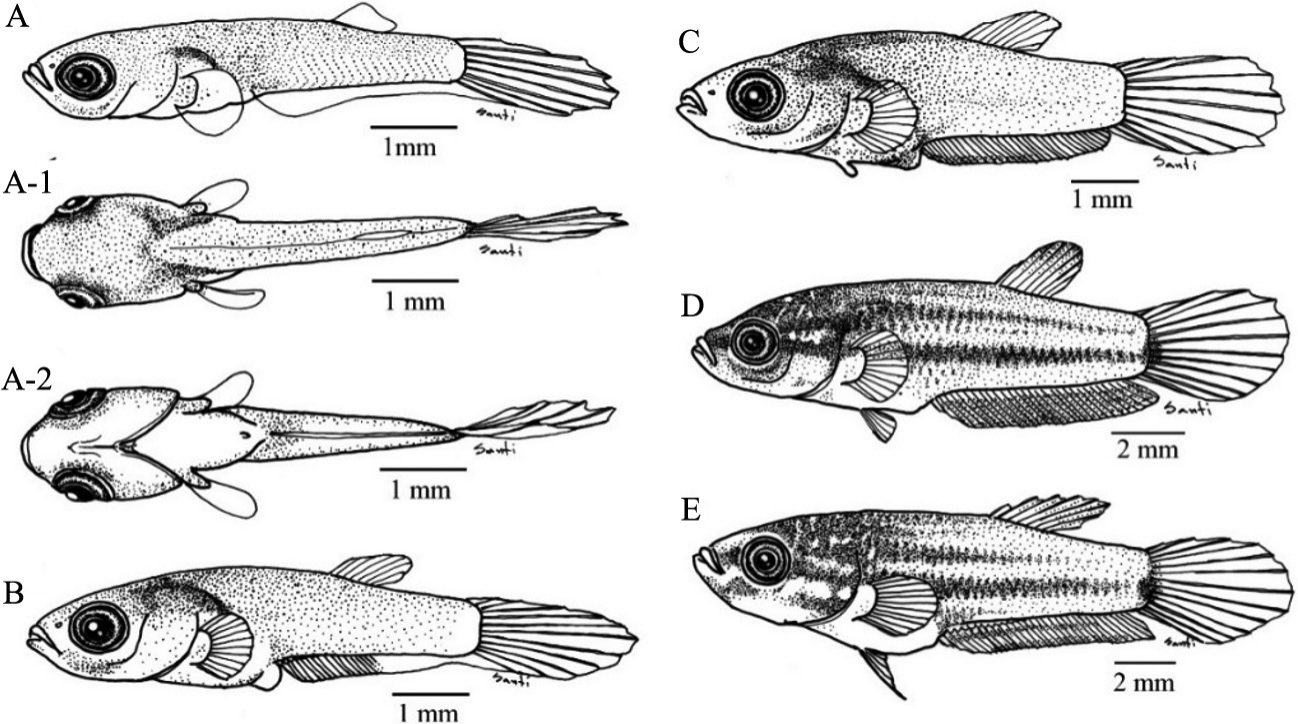
Morphology and pigmentation of *Betta apollon* Schindler & Schmidt, 2006 (KUMF 7782). **A**. First-release post-flexion larva (11 DAF) (SL = 4.76 mm); **B**. 3 DAR post-flexion larva (SL = 5.47 mm); **C**. 9 DAR post-flexion larva (SL = 6.09 mm); **D**. 18 DAR post-flexion larva (SL = 11.66 mm); **E**. 30 DAR juvenile (SL = 13.10 mm).

Upon first release, the post-flexion stage (11 DAF) (SL = 4.76 mm; Fig. 5A) had an oblong and slightly compressed posterior part of body (BD = 25.44%SL). Large and very depressed head (HL = 33.50%SL; HW = 123.81%HD), large and slightly oval eye (ED = 40.63%HL), head shape trapezoidal profile (HD_ant_ = 81.43%HD_post_). A small oblique mouth with a maxillary end tip beyond anterior edge of eye. Gut coiled and opened at midline of body (PAL = 50.00%SL). A total of 31 myomeres (*n* = 10), consisting of nine pre-anal and 22 post-anal myomeres, respectively. Small spots scattered along sub-orbital region, head, and antero-dorsal part of body beyond caudal fin base. Caudal fin ray is nearly complete. At the 3 DAR post-flexion stage (SL = 5.47 mm; Fig. 5B), eye develops to a rounded shape with ED = 39.74%HL, gut open at PAL = 55.44%SL. Pectoral, dorsal, and anal fin rays begin to develop. Pigmentation heavy dense on head and air-bladder regions. At the 9 DAR post-flexion stage (SL = 6.09 mm; Fig. 5C), completely developed pectoral, dorsal, anal, and caudal fin rays, while pelvic bud begins to develop. Pigmentation was heavy and dense in sub-orbital region and appeared as dorsal and central stripes on anterior part of body. At the 18 DAR post-flexion stage (SL = 11.66 mm; Fig. 5D), dorsal and central stripe extend to caudal fin base, light spots scattered around anal fin. At the 30 DAR juvenile stage (SL = 13.10 mm; Fig. 5E), pelvic fin ray was completely developed. Complete pigmentation on head was similar to adult stage, e.g., presence of pre-, sub-, first post-, and second post-orbital bands. Two longitudinal stripes presented, e.g., dorsal stripe and central stripe, which were separated from caudal spot. Fin ray count of juvenile specimens (*n* = 14): D = 9–10 (mode = 10), P_1_ = 10–11 (mode = 11), P_2_ = 6, A = 24–27 (mode = 25), and C = 10.

### *Betta ferox* (Schindler & Schmidt, 2006) (KUMF 7783)

**Figure 6.**
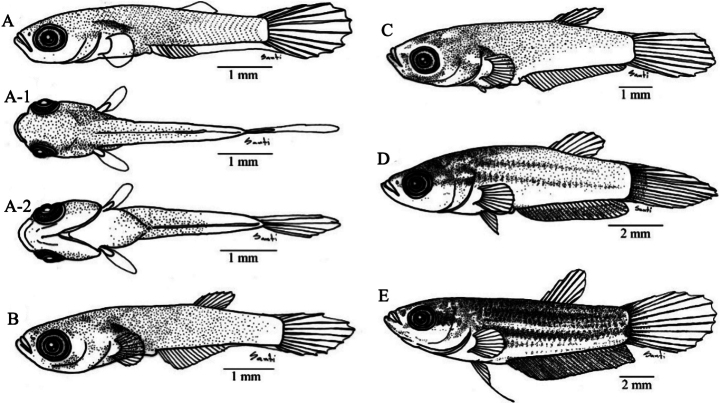
Morphology and pigmentation of *Betta ferox* (Schindler & Schmidt, 2006) (KUMF 7783). **A**. First-release post-flexion larva (11 DAF) (SL = 4.63 mm); **B**. 6 DAR post-flexion larva (SL = 5.09 mm); **C**. 14 DAR post-flexion larva (SL = 7.23 mm); **D**. 18 DAR post-flexion larva (SL = 8.91 mm); **E**. 30 DAR juvenile (SL = 13.52 mm).

Upon first release, post-flexion stage (11 DAF) (SL = 4.63 mm; Fig. 6A) had an oblong and slightly compressed posterior part of body (BD = 20.27%SL). Large and depressed head (HL = 32.43%SL; HW = 108.70%HD) with large and slightly oval eye (ED = 41.67%HL), head shape trapezoidal profile (HD_ant_ = 85.56%HD_post_). A small oblique mouth with maxillary end tip beyond anterior edge of eye. Gut coiled and opens slightly beyond the mid-body (PAL = 53.15%SL). A total of 31 myomeres (*n* = 6), consisting of ten pre-anal and 21 post-anal myomeres, respectively. Small spots scattered along head, except sub-orbital region, and antero-dorsal part of body beyond the dorsal fin base midline. Caudal fin ray nearly completely developed. At the 6 DAR post-flexion stage (SL = 5.09 mm; Fig. 6B), eye develops to a rounded shape with ED = 40.54%HL, and the gut opens at PAL = 51.54%SL. Pectoral, dorsal, and anal fin rays begin to develop. Pigmentation was dense in sub- and post-orbital regions. At the 14 DAR post-flexion stage (SL = 7.23 mm; Fig. 6C), completely developed pectoral, dorsal, anal, and caudal fin rays, while pelvic bud begins to develop. Scattered spots dense and reach the caudal peduncle. At the 18 DAR post-flexion stage (SL = 11.66 mm; Fig. 6D), heavy pigmentation appeared as dorsal and central stripes at anterior part of body. At the 30 DAR juvenile stage (SL = 13.10 mm; Fig. 6E), pelvic fin completely develops. Complete pigmentation on head was similar to adult stage, e.g., presence of pre- and first post-orbital and absences of sub- and second post-orbital bands. Two longitudinal stripes presented, e.g., dorsal stripe and central stripe, which connected to caudal spot. Fin ray count of juvenile specimens (*n* = 5): D = 8–9 (mode = 8), P_1_ = 11–12 (mode = 12), P_2_ = 6, A = 24–26 (mode = 25), and C = 10.

### *Betta pallida* Schindler & Schmidt, 2004 (KUMF 7784)

**Figure 7.**
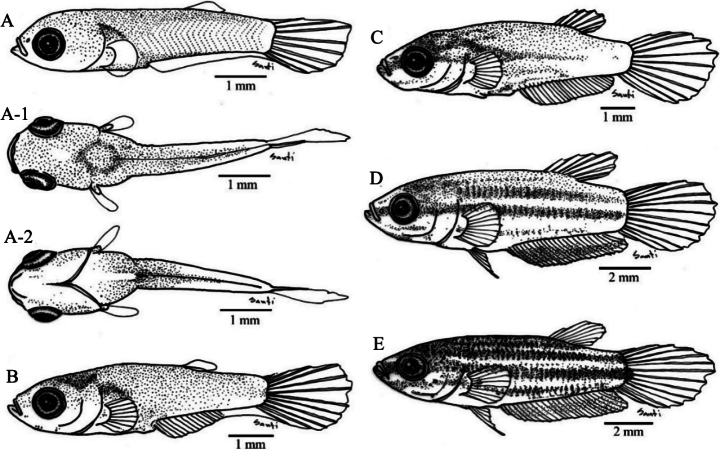
Morphology and pigmentation of *Betta pallida* Schindler & Schmidt, 2004 (KUMF 7784). **A**. First-release post-flexion larva (11 DAF) (SL = 4.98 mm); **B**. 6 DAR post-flexion larva (SL = 5.56 mm); **C**. 12 DAR post-flexion larva (SL = 7.62 mm); **D**. 18 DAR post-flexion larva (SL = 10.20 mm); **E**. 24 DAR juvenile (SL = 10.32 mm).

Upon first release, post-flexion stage (11 DAF) (SL = 4.98 mm; Fig. 7A) had an oblong and slightly compressed posterior part of body (BD = 23.53%SL). Large and slightly depressed head (HL = 33.33%SL; HW = 104.08%HD) with large and round eye (ED = 38.24%HL), rounded head in profile (HD_ant_ = 76.92%HD_post_). A small oblique mouth with a maxillary end tip beyond anterior edge of eye. Gut coiled and opens slightly beyond the midline of body (PAL = 51.98%SL). A total of 30 myomeres (*n* = 12), consisting of 9 pre-anal and 21 post-anal myomeres, respectively. Small spots scattered along head, except frontal and sub-orbital regions, and antero-dorsal part of body beyond the caudal peduncle. Caudal fin nearly complete. At the 6 DAR post-flexion stage (SL = 4.98 mm; Fig. 7B), completely developed caudal fin, while pectoral, dorsal, and anal fin rays begin to develop. Pigmentation was heavy and dense on air-bladder, ventral edge of gut, and lower edge of caudal region. At the 12 DAR post-flexion stage (SL = 7.62 mm; Fig. 7C), completely developed pectoral, dorsal, and anal fin rays, while pelvic bud begins to develop. Heavily pigmented and appear as central stripes in anterior part of body. At the 18 DAR post-flexion stage (SL = 10.20 mm; Fig. 7D), central stripe develops more beyond caudal fin base and appears as dorsal and central stripes in anterior part of body. At the 24 DAR juvenile stage (SL = 13.10 mm; Fig. 7E), pelvic fin completely develops. Complete pigmentation on head was similar to adult stage, e.g., presence of pre- and first post-orbital and absences of sub- and second post-orbital bands, central stripe that connected to caudal spot. Fin ray count of juvenile specimens (*n* = 16): D = 8–9 (mode = 8), P_1_ = 11–12 (mode = 12), P_2_ = 6, A = 26–28 (mode = 27), and C = 10–12 (mode = 11).

### *Betta pi* Tan, 1998 (KUMF 7788)

**Figure 8.**
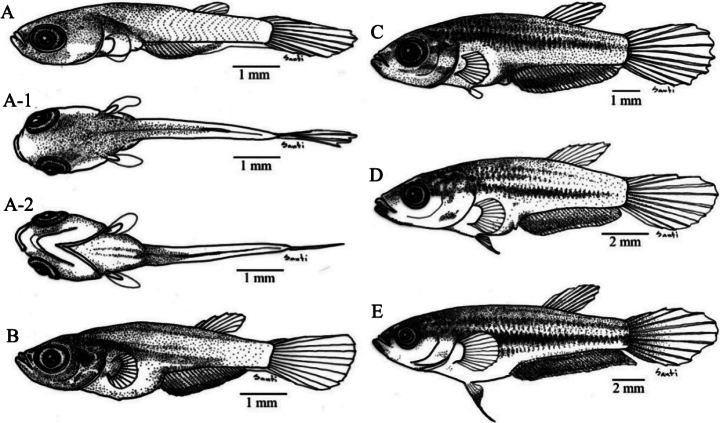
Morphology and pigmentation of *Betta pi* Tan, 1998 (KUMF 7788). **A**. First-release post-flexion larva (20 DAF) (SL = 5.60 mm); **B**. 3 DAR post-flexion larva (SL = 6.41 mm); **C**. 9 DAR post-flexion larva (SL = 9.48 mm); **D**. 13 DAR post-flexion larva (SL = 10.70 mm); **E**. 21 DAR juvenile (SL = 15.50 mm).

Upon first release, post-flexion stage (20 DAF) (SL = 5.60 mm; Fig. 8A) were oblong and slightly compressed in posterior part of body (BD = 23.53%SL). Large and very depressed head (HL = 33.48%SL; HW = 121.74%HD) with large and slightly oval eye (ED = 47.06%HL), conical head in profile (HD_ant_ = 81.82%HD_post_). A small oblique mouth with a maxillary end tip beyond anterior edge of eye. Gut coiled and opens slightly beyond mid-body (PAL = 49.11%SL). A total of 31 myomeres (*n* = 6), consisting of 11 pre-anal and 20 post-anal myomeres, respectively. Small spots scattered along head and anterior part of body beyond to dorsal fin base, two parallel stripes and a sub-marginal stripe along anal fin and ventral edge of belly. Caudal, dorsal, and anal fin rays develop. At the 3 DAR post-flexion stage (SL = 6.41 mm; Fig. 8B), completely developed caudal, dorsal, and anal fin rays, while pectoral fin begins to develop. Pigmentation was heavy and dense on head and anterior part of body, appeared on anterior part as central strip. At the 9 DAR post-flexion stage (SL = 9.48 mm; Fig. 8C), eye develops to a rounded shape, completely developed pectoral fin rays while pelvic bud begins to develop. Central stripes beyond caudal fin base. At the 13 DAR post-flexion stage (SL = 10.70 mm; Fig. 8D), anal fin ray develops, scattered spots on ventral side of head and body gradually fade. At the 21 DAR juvenile stage (SL = 15.50 mm; Fig. 8E), pelvic fin completely developed. Central stripe connects to caudal spot, and anterior part is approximately twice as deep as posterior part. Fin ray count of juvenile specimens (*n* = 5): D = 8-9 (mode = 8), P_1_ = 11–13 (mode = 11), P_2_ = 6, A = 30–31 (mode = 30), and C = 10–12 (mode = 11).

### *Betta prima* Kottelat, 1994 (KUMF 7780)

**Figure 9.**
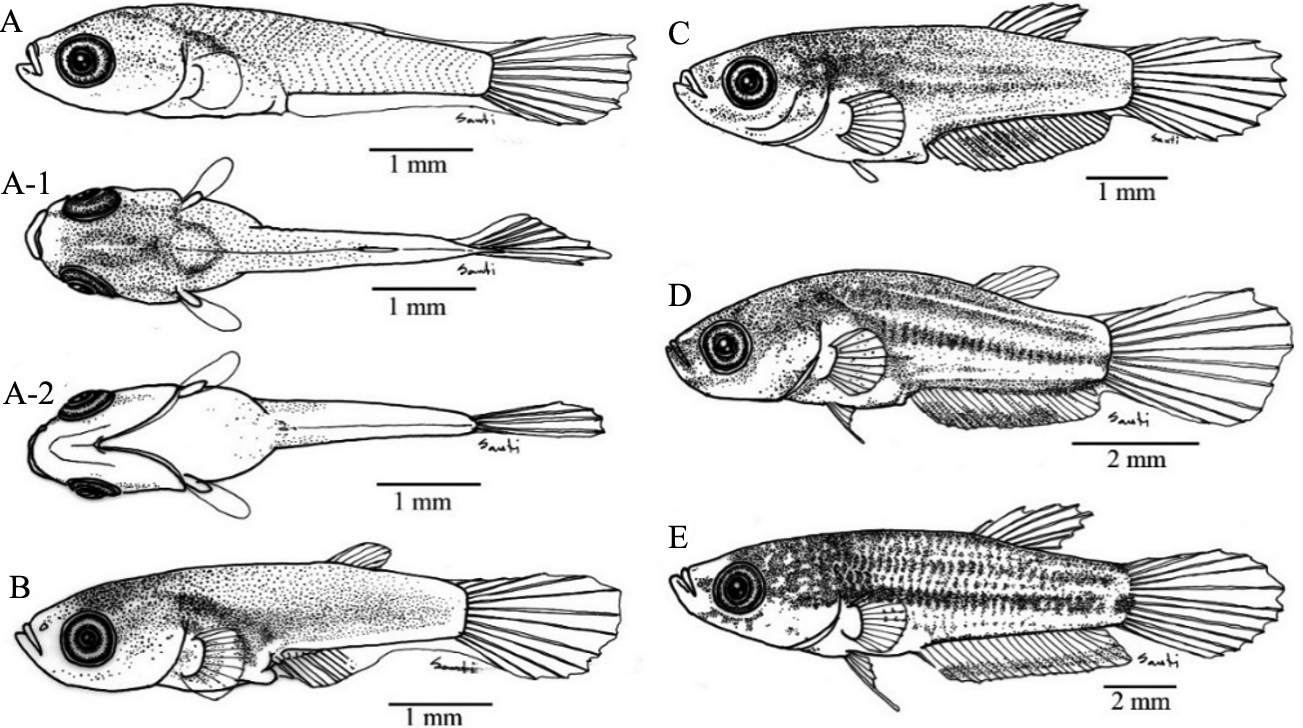
Morphology and pigmentation of *Betta prima* Kottelat, 1994 (KUMF 7780). **A**. First-release post-flexion larva (11 DAF) (SL = 3.73 mm); **B**. 6 DAR post-flexion larva (SL = 4.44 mm); **C**. 10 DAR post-flexion larva (SL = 5.37 mm); **D**. 18 DAR post-flexion larva (SL = 6.81 mm); **E**. 30 DAR juvenile (SL = 11.89 mm).

Upon first release, post-flexion stage (11 DAF) (SL = 3.73 mm; Fig. 9A) had an oblong and slightly compressed posterior part of body (BD = 25.44%SL). Moderate and slightly depressed head (HL = 32.35%SL; HW = 107.41%HD) with large and round eye (ED = 40.00%HL), rounded head in profile (HD_ant_ = 75.00%HD_post_). A small oblique mouth with a maxillary end tip not beyond anterior edge of eye. Gut coiled and opens slightly towards middle part of body (PAL = 54.39%SL). A total of 32 myomeres (*n* = 15), consisting of 11 pre-anal and 21 post-anal myomeres, respectively. Small spots scattered along head, including frontal region, and antero-dorsal part of body beyond dorsal fin base midline. Caudal fin was nearly completed. At the 6 DAR post-flexion stage (SL = 4.44 mm; Fig. 9B), a completely developed caudal fin, while pectoral, dorsal, and anal fin rays begin to develop. Pigmentation was heavily dense on air-bladder, pectoral fin base, and sub-ventral edge body region. At the 10 DAR post-flexion stage (SL = 5.37 mm; Fig. 9C), completely developed pectoral, dorsal, and anal fin rays while pelvic bud begins to develop. Heavily pigmented and appeared as central stripes at anterior part of body. At the 18 DAR post-flexion stage (SL = 6.81 mm; Fig. 9D), dorsal and central stipe developed more beyond caudal fin base, Ventral stripes appeared in anterior caudal part. At the 30 DAR juvenile stage (SL = 11.89 mm; Fig. 9E), pelvic fin completely developed. Complete pigmentation on head was similar to adult stage, central stripe that separates from caudal spot. Fin ray count of juvenile specimens (*n* = 12): D = 8–9 (mode = 8), P_1_ = 11–12 (mode = 12), P_2_ = 6, A = 25–27 (mode = 26), and C = 10-11 (mode = 11).

### *Betta pugnax* Cantor, 1840 (KUMF 7781)

**Figure 10.**
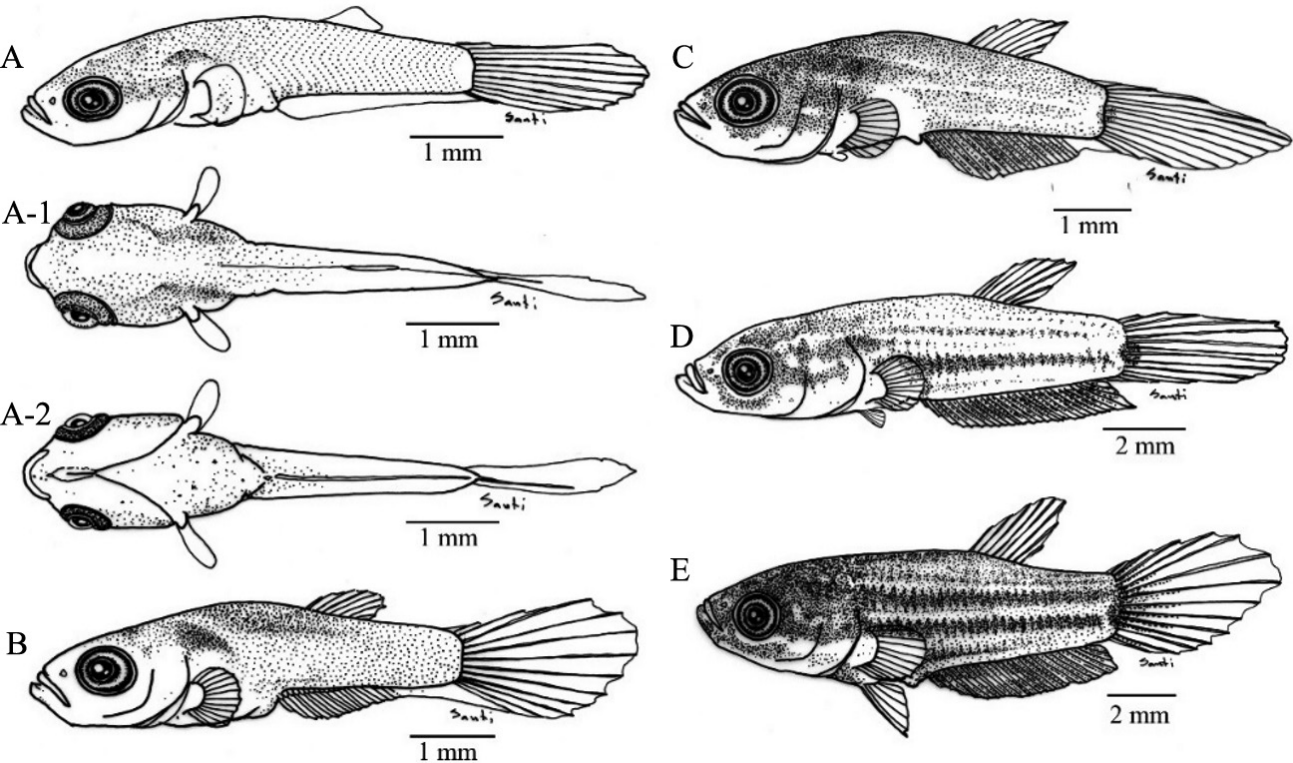
Morphology and pigmentation of *Betta pugnax* Cantor, 1840 (KUMF 7781). **A**. First-release post-flexion larva (11 DAF) (SL = 4.65 mm); **B**. 6 DAR post-flexion larva (SL = 5.10 mm); **C**. 10 DAR post-flexion larva (SL = 5.19 mm); **D**. 18 DAR post-flexion larva (SL = 8.91 mm); **E**. 30 DAR juvenile (SL = 12.00 mm).

**Figure 11.**
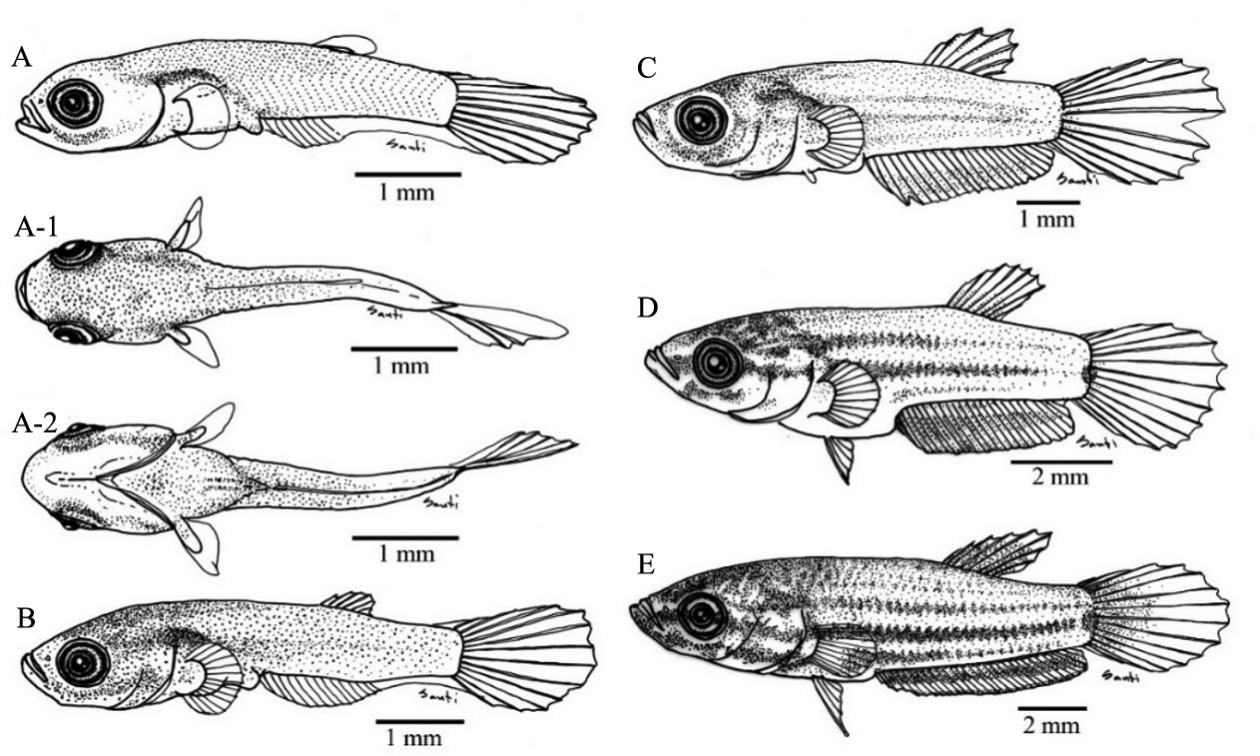
Morphology and pigmentation of *Betta simplex* Kottelat, 1994 (KUMF 7771). **A**. First-release post-flexion larva (11 DAF) (SL = 4.39 mm); **B**. 3 DAR post-flexion larva (SL = 4.86 mm); **C**. 9 DAR post-flexion larva (SL = 7.07 mm); **D**. 15 DAR post-flexion larva (SL = 8.56 mm); **E**. 30 DAR juvenile (SL = 11.68 mm).

Upon first release, post-flexion stage (11 DAF) (SL = 4.65 mm; Fig. 10A) had an oblong and slightly compressed posterior part of body (BD = 22.54%SL). Large and very depressed head (HL = 34.39%SL; HW = 134.09%HD) with large and oval eye (ED = 41.67%HL), trapezoidal head shape profile (HD_ant_ = 87.27%HD_post_). A small oblique mouth with a maxillary end tip beyond anterior edge of eye. Gut coiled and open at midline of body (PAL = 51.03%SL). A total of 31 myomeres (*n* = 9), consisting of nine pre-anal and 22 post-anal myomeres, respectively. Small spots scattered on head, except frontal- and sub-orbital region, and antero-dorsal part of body beyond dorsal fin base. Caudal fin ray nearly complete. At the 6 DAR post-flexion stage (SL = 5.10 mm; Fig. 10B), gut opened at PAL = 55.44%SL. Pectoral, dorsal, and anal fin rays begin to develop. Pigmentation was heavily dense on dorsal side of head and throughout body. At the 9 DAR post-flexion stage (SL = 6.09 mm; Fig. 10C), eye develops to a round shape. Pelvic bud begins to develop. Pigmentation was heavily dense in pre-, sub-orbital, first post-, and second post-orbital regions and appeared as dorsal, central, and ventral stipes. At the 18 DAR post-flexion stage (SL = 11.66 mm; Fig. 10D), central stripe extends to caudal fin base. At the 30 DAR juvenile stage (SL = 12.00 mm; Fig. 11E), pelvic fin ray completely developed. Complete pigmentation on head was similar to adult stage, e.g., presence of pre-, sub-, first post-, and second post-orbital bands, central stripe that separated from caudal spot. Fin ray count of juvenile specimens (*n* = 5): D = 9–10 (mode = 9), P_1_ = 11–12 (mode = 12), P_2_ = 6, A = 26–29 (mode = 27), and C = 10–12 (mode = 12).

### *Betta simplex* Kottelat, 1994 (KUMF 7771)

**Figure 12.**
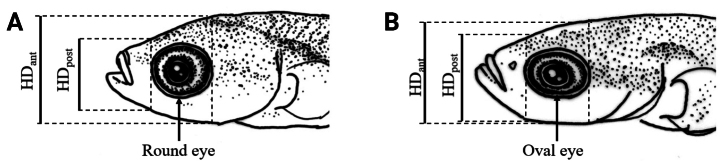
**A**. Round head (HDant < 80%HD post); **B**. Trapezoidal head (HDant > 80%HD post).

Upon first release, post-flexion stage (11 DAF) (SL = 4.39 mm; Fig. 12A) had an oblong and slightly compressed posterior part of body (BD = 19.68%SL). Moderate and depressed head (HL = 31.19%SL; HW = 115.23%HD) with large and round eye (ED = 41.18%HL), rounded head in profile (HD_ant_ = 90.74%HD_post_). Snout short, a small oblique mouth with a maxillary end tip part to anterior edge of eye. Gut coiled and opens slightly towards midline of body (PAL = 54.13%SL). A total of 31 myomeres (*n* = 9), consisting of 11 pre-anal and 20 post-anal myomeres, respectively. Small spots scattered along head, especially sub-orbital and second post-orbital region, and antero-dorsal part of body beyond dorsal fin base. Caudal fin nearly completed. At the 3 DAR post-flexion stage (SL = 4.86 mm; Fig. 12B), pectoral, dorsal, and anal fin rays begin to develop. Pigmentation was scattered throughout body and heavy and dense on antero-dorsal edge and air-bladder region. At the 9 DAR post-flexion stage (SL = 7.07 mm; Fig. 12C), completely developed pectoral, dorsal, anal, and caudal fin rays, while pelvic bud begins to develop. Dorsal, central, and ventral stripes begin to appear. At the 15 DAR post-flexion stage (SL = 8.56 mm; Fig. 12 Dd), central stripe develops more, beyond caudal peduncle. At the 30 DAR juvenile stage (SL = 13.10 mm; Fig. 12E), pelvic fin completely developed. Complete pigmentation on head was similar to adult stage, e.g., presence of pre-, sub- and post-orbitals, first post-orbital band connected to central stripes, central stripe separate from caudal spot, marginal and sub-marginal stripes in anal fin. Fin ray count of juvenile specimens (*n* = 12): D = 8–9 (mode = 8), P_1_ = 11–12 (mode = 12), P_2_ = 6, A = 24–25 (mode = 25), and C = 12–13 (mode = 12).

## Discussion

The environmental parameters of water quality results indicate that all seven wild mouth-brooding fighting fishes are adaptable to different rearing conditions. Generally, mouth-brooding fighting fishes are distributed in southern Thailand, except *B. prima*, which is found in eastern Thailand. The environmental conditions in the latter area create a diverse habitat for freshwater fish, including vegetative swamps, peat swamps, limestone streams, rivers, and rainforest creeks (Kottelat and Ng 1994). This environment is beneficial for fighting fish, particularly for the mouth-brooding species. Additionally, this area forms part of the Malay Peninsula and has been part of the Sundaic region for the last 2.6 million years. This region is highly diverse in labyrinth fishes (Roberts 1989; Kottelat and Ng 1994; Rüber et al. 2004). Some species live in specific conditions, such as *B. simplex* usually living in high-alkaline and hard limestone hill streams (Panitiwong 2020; Panijpan et al. 2020) and *B. pi* specifically living in cold and low-pH conditions of peat swamps (Schindler and Schmidt 2006). The fishes’ development to reproduction and spawning indicates their successful adaptation to these conditions. However, although mouth-brooding fighting fishes are able to breed in captivity, some environmental parameters should be considered and provided for better living quality. In this study, a water temperature of 26 °C was required for *B. pi* breeding, while pH was of no concern (Thai Agricultural Standard 2013). Living in favorable environmental conditions and with sufficient food would allow the fish to produce healthy offspring with high survival rates. Additionally, we note that in the case of early release of incubating eggs and offspring by the incubating male, although we tried to incubate them in an external incubation system, the egg and offspring became infected and died within 1–3 days. Yu et al. (2019) stated that some teleosts have oral immune systems, with protective substances secreted from a mucous gland under the oral epithelium, protecting the incubating eggs and offspring against pathogens within the external oral cavity.

New-release mouth-brooders have an expected age of 11 DAF and have developed to the post-flexion stage. These post-larvae have already absorbed their yolk sac, flexed up their notochords, part of their caudal fin rays, and developed their mesenchyme and dorsal and anal fin bases. When compared with a bubble-nester, *B. splendens*, as described by Poungcharean and Limpivadhana (2022), show that the mouth-brooders have a slower development at the same age. At day 9, *B. splendens* develops to the post-flexion stage with a smallest notochord length of 3.76 mm, while *B. pi* develops to the post-flexion stage with a smallest notochord length of 5.44 mm; the latter is the largest mouth-brooder and has a more extended incubation period (18–20 days) because of its large buccal cavity, beneficial for its offspring in a cold-water habitat. Additionally, Ashaf-Ud-Doulah et al. (2021) found that *Labeo rohita* presented low yolk sac absorption and long incubation times, including damaged embryos, at low temperatures. Mouth-brooders develop to the juvenile stage at different rates depending on their species. We note that the large species *B. pi* has a shorter development period (18 DAR) than the smaller species, especially *B. simplex* (30 DAR). Parental care of eggs and offspring in the mouth cavity is a behavioral adaptation of mouth brooders to running water conditions compared to the bubble nesters. Therefore, greater oral space, including head size, promotes more efficient care of eggs and offspring. Therefore, the mouth brooders have an increase in head size when they get older, with a head length of 31.18–34.45%SL for new release larvae vs 33.33–34.55%SL for juveniles, larger than the bubble nesters that have a head length less than 30.00%SL (Poungcharean and Limpivadhana 2022). Additionally, the head shape changes significantly with age, with new-release larvae having a depressed head with a head width of 104.08–134.09% HD, that gradually becomes compressed to a head width of 62.75–74.31% HD when they develop into juveniles.

The PCA testing of morphometric characters shows that the high loading factor of PC1 for head depth (0.9353) and PC2 for head width (0.8535) indicates significant variation in these characteristics, making them effective distinguishing characters for the new-release stages among the mouth-brooders. The PC1 axis of scatter plot of Fig. 4A and the range of the proportion of head width (HW) and head depth (HD) of Table 3 reflect the depressed head of each fish species. The overlapping of the minimal-maximal intervals differed in each species, aiding species identification. Also, the proportion of head depth characters of the PC2 axis, the head depth at anterior of eye (HD_ant_), and head depth at posterior of eye (HD_post_) can be used to distinguish the fish into two groups. The first group is the rounded head group, i.e., *B. pallida*, *B. prima*, and *B. simplex* that have HD_ant_ lower than 80%HD_post_ (HD_ant_ = 68.65–77.33%HD_post_). The other group is the trapezoidal head group, i.e., *B. apollon*, *B. ferox*, *B. pi*, and *B. pugnax* that have HD_ant_ higher than 80%HD_post_ (HD_ant_ = 81.37–87.34%HD_post_).

For meristic characters, the high loading factor on the PC1 axis for anal fin ray count (0.9512) allows for the distinction of *B. pi*, which has more anal fin rays (30 or 31) than other species with fewer anal fin rays (24–28). Although the PCA testing is able to differentiate some species from a group, it cannot be used for classification based on a single characteristic alone because the larval fish have fast growth rates and transformation. For classification of early-stage mouth-brooding fighting fishes, developmental stages should also be considered, particularly observing specimens with the characteristic flexed-up urostyle and beginning development of the dorsal and anal fin rays, which indicates the early post-flexion stage of bony fishes (Neira et al. 1998; Leis and Carson-Ewart 2000). Using a high loading factor of PC1 and the minimal-maximal interval of head length in Table 3 distinguishes *B. simplex* from the other rounded head species. The remaining species, *B. pallida*, was distinguished from *B. prima* by differences in the appearance of melanophore pigmentation on the dorsal body and frontal region. For the trapezoidal head group, our PCA morphometric characters were unavailable due to their low loading factor. Thus, the melanophore pigmentation could be considered a character at this stage. The presence of a marginal stripe on the anal fin of *B. pi* distinguishes it from other trapezoidal-headed species. The absence of any frontal pigmentation in *B. pugnax* distinguishes it from the remaining species. Finally, the presence of the sub-orbital and pectoral fin base band of *B. apollon* distinguished it from *B. ferox*, which lacks these characters.

In contrast, the appearance of pigmentation on the head among this group is inconsistent with the genetic classification of Panijpan et al. (2014), which separates *B. ferox* from the *B. pugnax* group (*B. apollon* and *B. pugnax*). Our study, *B. pugnax* is separated by the absence of pigmentation in the frontal region from *B. apollon* and *B. ferox*, which have pigmentation. Morphometric and meristic characteristics cannot distinguish the *B. prima* group (*B. pallida* and *B. prima*) due to their similarities and overlap in data. The pigmentation on the dorsal side of the body can only be used to identify the new release flexion stage, while the head and body bands are relevant for the juvenile stage. We believe that these fishes were connected to the same river basin system during the formation of Sundaland that then diverged into two populations due to rising sea levels after the end of the ice age, evolving into new species, i.e., *B. prima* in the eastern region and *B. pallida* in the western region of the former Sundaland (Roberts and Jumnongthai 1999; Šlechtová et al. 2021).

The new-release mouth-brooders in this study and the 9–12 DAF bubble-nesters reported by Poungcharean and Limpivadhana (2022) and Termvidchakorn (2005) are both the same age and have developed to the post-flexion stage, sharing characteristics such as an oblong body and similar head and body profiles. However, there are some notable differences between them, such as their proportional head length (28.12–36.42 vs 24.14–28.31%SL), head width (> 100.00 vs < 85.00% HD), and pigmentation patterns on the dorsal side of the head and anterior part of the body (absence vs presence of dorsal and central stripe). Additionally, the myomere count was a second distinguishing character, even though there are overlapping ranges (30–32, mode = 30, vs 29–31, mode = 29, respectively). At the juvenile stage, the two groups can be distinguished by fin ray count, as well as at the adult stage, which was compared and described by Kowasupat et al. (2012), Panijpan et al. (2017) and Rüber et al. (2004). We found that the character of fin ray count assists identification for some species in the juvenile stage, such as the > 30 anal fin rays of *B. pi* compared to other species, which have < 29 anal fin rays. Additionally, the various juvenile stages exhibited differences in pigmentation patterns among species, including specific patterns on the head (pre-orbital, sub-orbital, post-orbital, and sub-opercular bands) and longitudinal stripes along the sides of the body and anal fin that are useful. For the juvenile stage identification, the anal fin count is the only meristic character available. The high loading factor PC1 distinguishes the greater number of anal fin rays of *B. pi* from the other species. This finding is consistent with the description of the adult stage reported by Kottelat (1984), Kottelat and Ng (1994), and Schindler and Schmidt (2006). However, this study focused only on the total number of fin rays without classifying them as spinous or soft fin rays, as this is a difficult characteristic to observe and requires clarification for their further development.

In this study, we use the classification systems of Schindler and Schmidt (2006) as the primary taxonomic basis, including generating the identification keys. Fin ray count was the first ordering characteristic, followed by body proportion and melanophore pigmentation for generating dichotomous keys, according to Poungcharean and Limpivadhana (2022) and Termvidchakorn (2005). But, due to the limitation of the information on fin ray count and body proportion traits, it is difficult to identify the fighting fishes, which are very similar at species level. Therefore, it indicated that the quantitative data is not beneficial to species-level identification for the mouth-brooding fighting fishes at this stage. The pattern of head, body, and anal fin pigmentation was the best alternative trait as a distinguishing character for species-level identification in both the newly released post-flexion and juvenile stages. Thus, further studies should confirm these fishes to species level by combining them with molecular techniques. Based on the descriptions and illustrations of the seven mouth-brooders, separate dichotomous keys could be generated for the post-flexion stage (new release) and juvenile stage (18–30 DAR).

### Key for post-flexion stage of mouth-brooding fighting fishes (new-release)

**Table:** 

1	Round head, HD_ant_ lower than 80%HD_post_, round eye (Fig. 12A)	**2**
–	Trapezoidal or conical head, HD_ant_ higher than 80%HD_post_, oval eye (Fig. 12B)	**4**
2	Short head, HL lower than 31.21%HL, sub-orbital band and pigmentation in antero-ventral region of body present (Fig. 13A)	***B. simplex***
–	Long head, HL higher than 32.33%HL, Sub-orbital band and pigmentation in antero-ventral region of body absent (Fig. 13B)	**3**
3	Scattered spots on dorsal body beyond dorsal fin base, pigmentation in frontal region (Fig. 14A)	***B. prima***
–	Scattered spots on dorsal body beyond caudal peduncle, no pigmentation in frontal region (Fig. 14B)	***B. pallida***
4	Marginal stripe in anal fin present (Fig. 15A)	***B. pi***
–	Marginal stripe in anal fin absent (Fig. 15B)	**5**
5	No pigmentation in frontal region (Fig. 16A)	***B. pugnax***
–	Pigmentation in frontal region present (Fig. 16B)	**6**
6	Sub-orbital band present, pigmented in pectoral fin base (Fig. 17A)	***B. apollon***
–	Sub-orbital band absent, no pigmentated in pectoral fin base (Fig. 17B)	***B. ferox***

**Figure 13.**
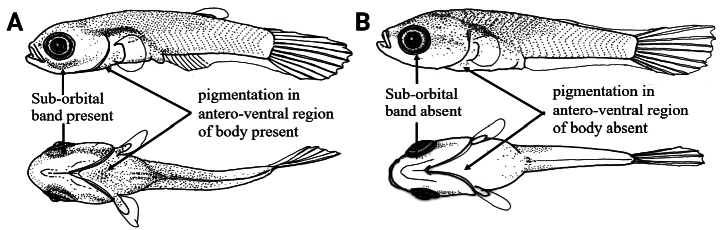
**A, B**. *B. simplex*.

**Figure 14.**
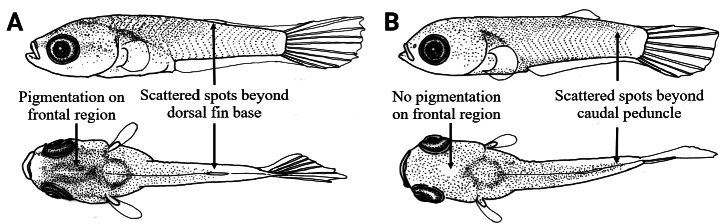
**A**. *B. prima*; **B**. *B. pallida*.

**Figure 15.**
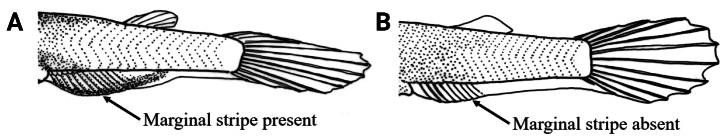
**A, B**. *B. pi*.

**Figure 16.**
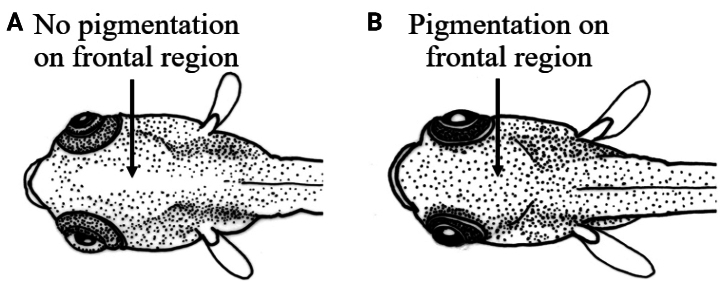
**A, B**. *B. pugnax*.

**Figure 17.**
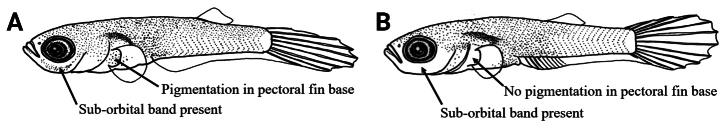
**A**. *B. apollon*; **B**. *B. ferox*.

### Key for juvenile stage of mouth-brooding fighting fishes (21-30 DAR)

**Table:** 

1	Sub-orbital band absent, anterior of central stripe deeper than posterior, ≥ 30 anal fin rays (Fig. 18A)	***B. pi***
–	Sub-orbital band present, anterior of central stripe depth equal to posterior, ≤ 29 anal fin rays (Fig. 18B)	**2**
2	Sub-orbital band connects to second post-orbital band, anal fin rear tip rounded and beyond caudal fin base (Fig. 19A)	***B. simplex***
–	Sub-orbital band separates from second post-orbital band, anal fin rear tip pointed and extend to caudal fin base (Fig. 19B)	**3**
3	First sub-marginal stripe at anal fin edge present and second sub-marginal stripe absent (Fig. 20A)	**4**
–	Marginal stripe or second sub-marginal stripe at anal fin edge present (Fig. 20B) or pigmentation covers entire anal fin (Fig. 20C)	**5**
4	Second post-orbital and pectoral base band absent, central stripe separates from caudal spot (Fig. 21A)	***B. prima***
–	Second post-orbital and pectoral base band present, central stripe connects to caudal spot (Fig. 21B)	***B. pallida***
5	Marginal stripe, sub-marginal stripe at anal fin, second post-orbital band present (Fig. 22A)	***B. apollon***
–	Second post-orbital band and marginal stripe at anal fin edge fade or absent	**6**
6	Heavily sub-orbital band, second post-orbital band fade (Fig. 22B)	***B. pugnax***
–	Lightly sub-orbital band, second post-orbital band absent (Fig. 22C)	***B. ferox***

**Figure 18.**
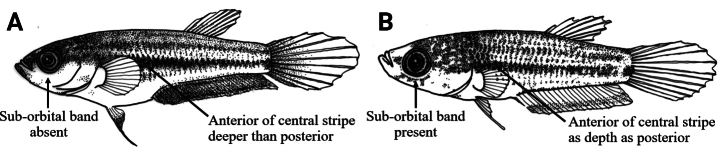
**A, B**. *B. pi*.

**Figure 19.**
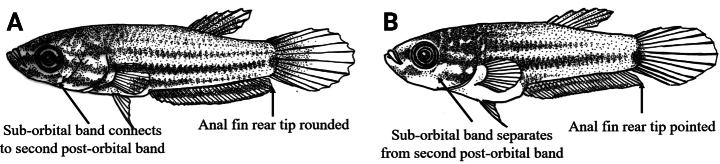
**A, B**. *B. simplex*.

**Figure 20.**
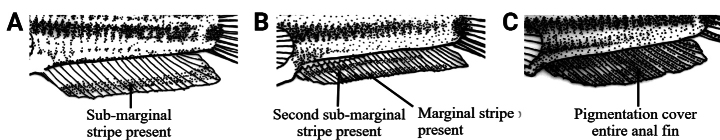
**A, B, C**. *B. simplex*.

**Figure 21.**
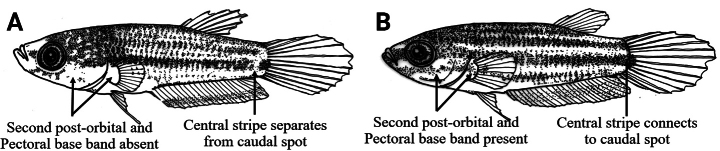
**A**. *B. prima*; **B**. *B. pallida*.

**Figure 22.**
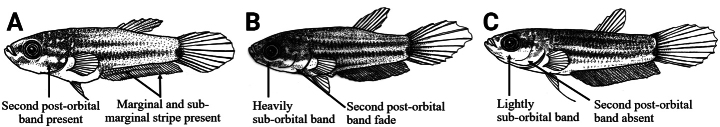
**A**. *B. apollon*; **B**. *B. pugnax*; **C**. *B. ferox*.

## Conclusions

Six wild mouth-brooding fighting fishes, i.e., *Betta apollon*, *B. ferox*, *B. pallida*, *B. pi*, *B. prima*, and *B. pugnax*, were successfully bred in captivity. All of their offspring were collected for producing a reference size series, and *B. simplex* was described for morphological development. The useful characteristics for identifying mouth-brooders at species level were the number of anal fin rays, the proportions of head and body, and melanophore pigmentation on the head, body, and fins.

## Additional information

### Artificial Intelligence (AI) use

The authors accept full responsibility for the content of the manuscript, including the disclosure of any use of AI.

No AI tools were used in the preparation of this manuscript.

### Author ORCIDs

S. Poungcharean https://orcid.org/0000-0002-8521-4897

I. Wudtisin https://orcid.org/0000-0001-7840-2964

S. Sirisuay https://orcid.org/0000-0002-6641-8384

P. Pichitkul https://orcid.org/0000-0002-0458-8951

S. Janekitkarn https://orcid.org/0000-0002-4002-6729

### Data availability

All of the data that support the findings of this study are available in the main text.
